# Effects of Rearing Conditions and Sex on Cecal Microbiota in Ducks

**DOI:** 10.3389/fmicb.2020.565367

**Published:** 2020-10-08

**Authors:** Chunhong Zhu, Wenjuan Xu, Zhiyun Tao, Weitao Song, Hongxiang Liu, Shuangjie Zhang, Huifang Li

**Affiliations:** Jiangsu Institute of Poultry Science, Yangzhou, China

**Keywords:** rearing conditions, sex, cecum, microbiota, duck

## Abstract

The intestinal microbiome influences the health of animals. However, little is known about the impact of indoor conditions and sex on intestinal microbiome diversity and composition in ducks. The present study aimed to investigate differences in the cecal microbiome between male and female ducks reared on the floor (PY group) or in cages (LY group). We also determined the relationships between cecal microbiota composition and slaughter traits, and the expression levels of mucosal and intestinal structural genes in ducks. There was a slight difference in slaughter traits among the groups, with cecum weight being significantly lighter in the LY compared with the PY group, especially in females (*p* < 0.05). Analysis of the alpha diversity of the cecal microbiota between males and females in the LY and PY groups showed that LY males had significantly lower diversity and richness. Beta diversity analysis demonstrated differences in the microbiota composition in relation to rearing conditions, and a significant difference between the sexes in the PY groups. The dominant bacterial phyla in duck cecum were Bacteroidetes, Firmicutes, Proteobacteria, and Fusobacteria. The relative abundances of the most common bacteria revealed that the intestinal microbiota diversity and composition were affected by both feeding conditions and sex. Several bacterial genera were detected differentially among the groups. These genera were correlated with slaughter traits and expression levels of mucosal and cecal structural genes in ducks. In conclusion, rearing conditions, sex, and associated changes in the cecal microbiota are thus associated with gut barrier functions in ducks.

## Introduction

The intestinal tract is inhabited by numerous microorganisms, collectively referred to as the intestinal microbiota, most of which have a mutualistic relationship with their host (Schroeder and Bäckhed, 2016). The intestinal microbiota has received increasing attention in recent years, and has been shown to affect the structure and function of the intestine, increase the energy harvested from the diet, and drive the development of the immune system (McFall-Ngai et al., 2013). Even domestic animals with genetically similar backgrounds from the same producer may have different intestinal microbiota, depending on the conditions in where they are housed and their feeding patterns, suggesting that the environment and treatment are major factors in determining the intestinal microbiota (Friswell et al., 2010). Previous reports have demonstrated significant changes in the intestinal microbiota in response to housing conditions in mice and other animals, further corroborating the importance of the living environment in shaping the intestinal microbiota (Zhao et al., 2013; Pan and Yu, 2014; Ericsson et al., 2018).

Several studies have reported on the impact of housing conditions and feeding patterns on the health and meat quality of poultry, including chickens and ducks (Almasi et al., 2015; Yilmaz Dikmen et al., 2016). However, information on the effects of feeding patterns on the intestinal environment and the composition of the intestinal microbiota is still lacking. To the best of our knowledge, only some studies have examined the effect of rearing conditions on the intestinal microbiota in chickens or ducks (Xu et al., 2016; Wang et al., 2018).

The intestinal microbiota are separated from the host by a single layer of enterocytes and other barriers, including intestinal mucus and a continuous epithelial layer (Schroeder, 2019). Mucin 2 is the main structural component of the mucus layer (Lindén et al., 2008), while occludin (Furuse et al., 1993) and claudins (Furuse et al., 1998) are important tight junction transmembrane proteins involved in the formation of a continuous layer of epithelial cells. However, the role of the structure and composition of the intestinal microbiota on the intestinal mucus layer and structure in ducks remains unclear. Furthermore, some studies have suggested the existence of sex-dependent effects on the intestinal microbiota in various animal models (McKenna et al., 2008; Yurkovetskiy et al., 2013; Bolnick et al., 2014; Borda-Molina et al., 2020). Under normal circumstances, males grow faster than females, probably due to sexual differences in growth and development (Osei-Amponsah et al., 2012; Benyi et al., 2015). Thus, we hypothesized that these sexual variations in growth rate may be associated with sex-related differences in the intestinal microbiota.

It is crucial to understand how rearing conditions might impact the gut microbiomes and their subsequent impact on host traits for animal welfare and production. Thus, in this study we anticipate improving our knowledge on microbial changes associated with rearing condition through investigating the cecal microbiomes of ducks. The cecum plays important physiological functions, especially in poultry. It has a lower pH and higher content of easily fermentable compounds in chickens and ducks (Rojas-Feria et al., 2018), and cecal microbiomes has a considerable effect on nutrient digestion, absorption, and metabolism (Turnbaugh et al., 2006; Rinttilä and Apajalahti, 2013). In this study, we hypothesized that different feeding conditions might be related to the diversity and composition of the cecal microbiome, and that this microbiota might differ between male and female ducks under similar housing conditions. The results of this study will provide important information to support duck production and welfare.

## Materials and Methods

### Animal Feeding and Management

This study was conducted at Jiangsu Gaoyou Duck Co., Ltd. (Jiangsu, China). Two thousand Gaoyou ducks (1000 males, 1000 females) were fed on the plastic mesh floor for 100 days, after which 1000 ducks were transferred to A-type cages with one duck per cage in one shed (LY group), and the other 1000 ducks were fed on the floor (5 replicates) in another shed with access to an indoor water pool (about 4 h in the pool each day, with water change every 2 days), at a flock density of 8 ducks per square meter (PY group). These ducks were fed ad libitum with the same commercial formula diet, which mainly contained corn, soya bean meal, and wheat-middling, and which conformed to National Research Council nutrient recommendations (1994). The ducks were healthy and received no antibiotic treatments during the experiment. The ducks were fed in the two groups for 200 days.

The animal experiments were approved by the Committee of Animal Care at Jiangsu Institute of Poultry Science (CAC-JIPS01453, Yangzhou, China).

### Animal Slaughter and Sample Collection

Ten males and 10 females from each group were selected at random, weighed, and sacrificed after fasting for 8 h. Slaughtering was carried out according to the standard NY/T 823-2004 (Ministry of Agriculture and Rural Affairs, China). Slaughter traits included dressed weight, dressed percentage, and percentages of half-eviscerated yield, eviscerated yield, leg muscle, breast muscle, and abdominal fat.

Cecal contents were collected into 5 mL cryopreservation tubes and stored immediately at −80°C for further analysis. The cecum was weighed and sampled for RNA extraction and real-time fluorescence quantitative polymerase chain reaction (qPCR).

### Real-Time Fluorescence qPCR

We designed qPCR primers (Table 1) for the mucosal gene *MUC2* (mucin 2) and the intestinal structural genes *OCLN* (occludin), *CLDN1* (claudin 1), and *CLDN2* (claudin 2). Gene expression levels were calculated using the ΔΔ^*Ct*^ method using β-actin as the reference gene.

**TABLE 1:** Primers for real-time fluorescence qPCR.

**Gene name**	**Primers**	**Accession no.**
*MUC2*	F: 5′- GTCAGTCATGGTGGCCGTGTAAC-3′	XM_005024513.3
	R: 5′- CGTCATCAAGGACTTGCACAGGAG-3′	
*OCLN*	F: 5′- ATGACCGGCGGCTACTACTACAG-3′	XM_013109403.2
	R: 5′- GAAGCAGATGAGGCAGAGCAAGAG-3′	
*CLDN1*	F: 5′- GACCAGGTGAAGAAGATGCGGATG′	XM_013109403.2
	R: 5′- CGAGCCACTCTGTTGCCATACC-3′	
*CLDN2*	F: 5′- CCGACAGCACCAAGTACGAGATGG-3′	XM_005009661.3
	R: 5′- GCAGAGGATGAAGCCACCGATG-3′	
β-actin	F: 5′-TGAGAGTAGCCCCTGAGGAGCAC-3′	EF667345.1
	R: 5′-TAACACCATCACCAGAGTCCATCAC-3′	

Total mRNA were prepared from cecum tissue and first-strand cDNA was synthesized using Superscript II Reverse Transcriptase (Invitrogen, Carlsbad, United States). qRT-PCR was carried out with Super Real PreMix (SYBR Green) (FP204-01) master mix on a Real-Time PCR Detection System (Tiangen Biotech Co., Ltd., Beijing, China) and an ABI PRISM 7900 Sequence Detection System (Applied Biosystems, Waltham, United States) using the following program: 95°C for 10 min; 45 cycles of 95°C for 10 s, 60°C for 10 s, 72°C for 10 s, and 72°C for 6 min.

### DNA Extraction, Amplification, and Sequencing

Ten DNA samples were extracted from the cecal contents for each group. Total genomic DNA was extracted using the cetyltrimethylammonium bromide method (Tang et al., 2008). The quality and quantity of the DNA was verified using a NanoDrop^TM^ 2000 spectrophotometer (Thermo Scientific, MA, United States) and agarose gel electrophoresis. The extracted DNA was diluted to a concentration of 1 ng/μL and stored at −20°C. The diluted DNA was then used as a template for PCR amplification of the bacterial 16S rRNA genes, using barcoded primers and HiFi HotStart ReadyMix (KAPA Biosystems, MA, United States). For bacterial diversity analysis, the V3–V4 variable regions of the 16S rRNA gene was amplified using the universal primers 343F and 798R (343F: TACGGRAGGCAGCAG; 798R: AGGGTATCTAATCCT). Amplicon quality was checked by visualization with gel electrophoresis, followed by purification using AMPure XP beads (Agencourt, CA, United States). Equal amounts of purified amplicons were pooled for subsequent sequencing.

### Bioinformatics Analysis

Raw sequencing data were obtained in the FASTQ format. Paired-end reads were preprocessed using Trimmomatic software (Bolger et al., 2014) to remove ambiguous bases (N) and low-quality sequences (average quality score < 20), using a sliding-window trimming approach. After trimming, the paired-end reads were assembled using FLASH software with the following parameters: minimal overlap 10 bp, maximum overlap 200 bp, and maximum mismatch rate 20% (Reyon et al., 2012). The sequences were further de-noised using QIIME software (version 1.9.1) (Caporaso et al., 2010), and all sequences with bases > Q20 were retained. The reads were compared with the reference database (Silva database, https://www.arb-silva.de/) (Quast et al., 2013) using the UCHIME algorithm^1^ (Edgar et al., 2011) to detect and remove chimeric sequences (Haas et al., 2011).

The primer sequences were removed and the clean reads were finally obtained. The clean reads were then clustered to generate operational taxonomic units (OTUs) using Vsearch software, with a similarity cutoff of 97% (Edgar, 2013). A representative read for each OTU was selected using the QIIME package. All representative reads were annotated and searched with BLAST against the Silva database release 132 using the Ribosomal Database Project classifier (confidence threshold of 80%) (Wang et al., 2007).

The alpha diversity metrics (observed species and shannon’s index) were calculated using QIIME software (version 1.9.1) and displayed with the R package (version 2.15.3). Differences in the alpha diversity indices were analyzed with Wilcoxon’s test using the agricolae package in R software (version 2.15.3). For beta diversity metrics, unweighted UniFrac distance matrices were calculated using QIIME software (version 1.9.1). The ANOSIM non-parametric procedure was used to test for significant difference among groups. Principal components analysis (PCoA) figures were generated based on the FactoMineR package and ggplot 2 package in R software (version 2.15.3). Linear discriminant analysis effect size (LEfSe) analysis was performed using LEfSe software, and the screening value (the linear discriminant analysis (LDA) score) was 3. The Benjamini and Hochberg false discovery rate was used to revise the *p* values (White et al., 2009). Correlations between the bacterial genera in the cecum and slaughter traits and expression levels of structural cecal genes were investigated using Pearson’s correlation analysis, with *p* < 0.05 was considered to indicate a significant correlation.

### Statistical Analysis

Bodyweight, cecum weight, and slaughter traits were assessed by analysis of variance (ANOVA). Differences in the relative abundances of bacterial phyla and genera between groups were analyzed with the Mann–Whitney U test. All analyses were performed using IBM SPSS v. 20.0 (SPSS Inc., Chicago, IL, United States). For all tests, *p* < 0.05 was considered statistically significant.

## Results

### Slaughter Traits in Ducks With Different Feeding Conditions

The slaughter indexes in ducks from different groups are shown in Table 2. Male ducks were significantly heavier than females (*p* < 0.05) in the PY group, but there was no difference between males and females in the LY group. Body weights were higher in the LY compared with the PY group.

**TABLE 2:** Slaughter indexes for in the PY and LY groups.

**Indexes**	**PY group**		**LY group**	
	**Males**	**Females**	**Males**	**Females**
Body weight/g	2207.8 ± 42.015^a^	2052.4 ± 39.158^b^	2243.8 ± 39.254^a^	2134.5 ± 50.232^ab^
Percentage of half-eviscerated yield /%	0.824 ± 0.009	0.842 ± 0.010	0.828 ± 0.007	0.839 ± 0.008
Percentage of eviscerated yield /%	0.734 ± 0.008	0.753 ± 0.011	0.739 ± 0.007	0.752 ± 0.007
Dressed percentage /%	0.923 ± 0.008^ab^	0.932 ± 0.005^a^	0.905 ± 0.006^b^	0.917 ± 0.006^ab^
Percentage of breast muscle /%	0.121 ± 0.003^a^	0.128 ± 0.003^a^	0.110 ± 0.002^b^	0.109 ± 0.002^b^
Percentage of leg muscle /%	0.105 ± 0.003	0.101 ± 0.003	0.098 ± 0.002	0.104 ± 0.004
Percentage of abdominal fat /%	0.020 ± 0.001^b^	0.024 ± 0.002^ab^	0.027 ± 0.002^a^	0.029 ± 0.002^a^
Cecum weight/g	3.093 ± 0.198^a^	2.673 ± 0.124^ab^	2.521 ± 0.103^b^	2.389 ± 0.214^b^

*PY group, ducks reared on floor, LY group, ducks reared in A-type cages. N = 10,Different letters in the same row indicate significant difference among groups (*p* < 0.05), same letters in the same row indicate no significant difference among groups (*p* > 0.05).*

There was no significant difference in the percentage of half-eviscerated yield and percentage of eviscerated yield among the different groups. The dressed percentage was lower in the LY compared with the PY group, and was significantly lower in males in the LY group compared with females in the PY group (*p* < 0.05). The percentage of breast muscle was significantly higher in the PY group (*p* < 0.05) than the LY group, while the percentage of abdominal fat was significantly lower in the PY group compared with the LY group, especially for males in the PY group (*p* < 0.05).

Cecal weights were significantly lighter in the LY group, especially in females, compared with the PY group (*p* < 0.05).

### Expression of Cecum Mucosal and Structural Genes

**FIGURE 1:**
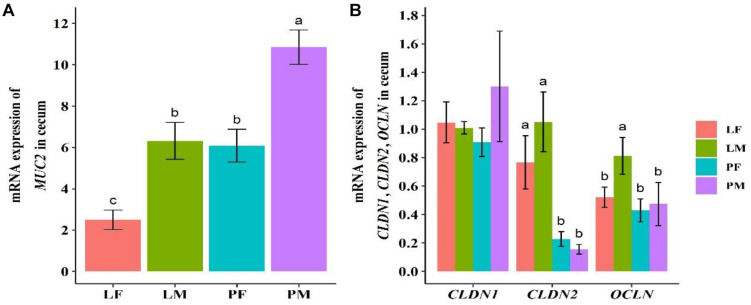
Expression levels of mucosal gene *MUC2* **(A)** and intestinal structural genes *OCLN*, *CLDN1*, and *CLDN2* **(B)** in cecal tissue in ducks. PF, females reared on floor; PM, males reared on floor; LF, females reared in A-type cages; LM, males reared in A-type cages. Different letters indicate significant difference among groups (*p* < 0.05), same letters indicate no significant difference among groups (*p* >0.05). *N* = 10.

The mucosal gene *MUC2* and the intestinal structural genes *OCLN*, *CLDN1*, and *CLDN2* in the cecum were detected by real-time fluorescence qPCR (Figure 1). *MUC2* expression was significantly higher in male ducks reared on the floor (PM) group, compared with female ducks reared in A-type cages (LF) group (*p* = 0.001). There was a significant difference between the sexes in both group (*p* < 0.05). *OCLN* and *CLDN2* showed similar trends in the PY and LY groups, and expression levels of *CLDN2* was significantly higher in the LY group (both males and females) compared with the PY group (*p* < 0.05 or 0.01). Expression levels of *OCLN* also differed significantly between males and females in the LY group, but there was no significant difference between the sexes in the PY group. There was no difference in *CLDN1* expression levels among the four groups.

### Alpha Diversity Analysis

**FIGURE 2:**
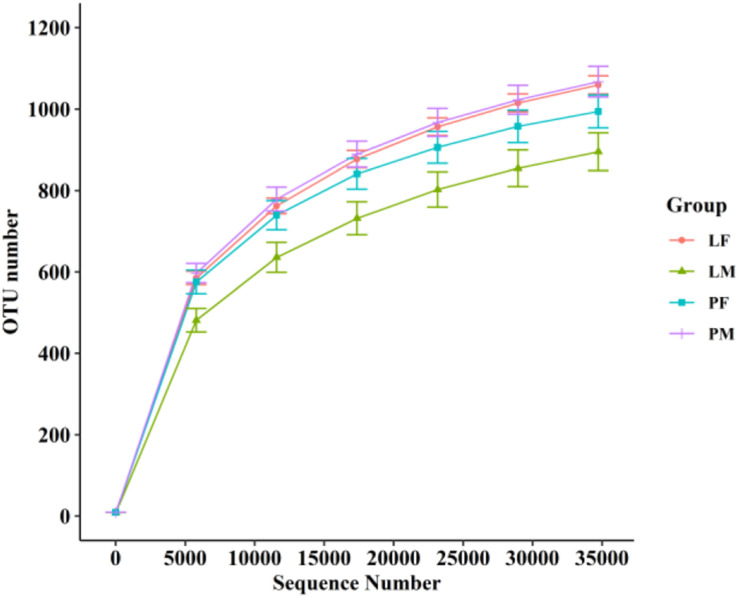
Rarefaction curves of PF, PM, LF, and LM groups. PF, females reared on floor; PM, males reared on floor; LF, females reared in A-type cages; PF, males reared in A-type cages.

**FIGURE 3:**
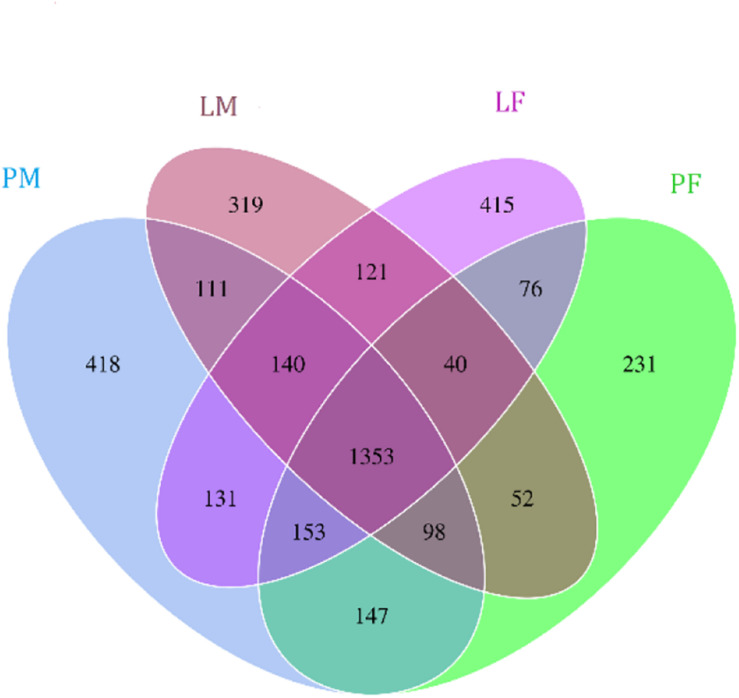
Flower plots of cecal microbiota in male and female ducks reared on the floor and in cages (based on OTUs). Each circle in the Venn diagram represents one group noted by same color. Numbers in the overlapping areas represent the number of bacterial OTUs shared between the respective groups. Numbers in the individual areas represent the number of bacterial OTUs exclusive to that group. PF, females reared on floor; PM, males reared on floor; LF, females reared in A-type cages; LM, males reared in A-type cages.

A total of 3805 bacterial OTUs were detected, including 2429, 2234, 2150, and 2551 in the LF, LM (male ducks reared in A-type cage), PF (female ducks reared on floor) and PM groups, respectively. Rarefaction curves generated from the OTUs suggested that high sampling coverage was achieved in all groups (Figure 2). These four groups shared 1353 bacterial OTUs. The cecal contents included different numbers of bacterial OTUs in relation to rearing conditions and sex. A total of 418 bacterial OTUs were uniquely sequenced in PM, compared with 231 in PF, and 319 bacterial OTUs were uniquely sequenced in LM, compared with 415 in LF (Figure 3). More bacterial OTUs were detected in female ducks in the LY group, but conversely, more bacterial OTUs were detected in male ducks in the PY group.

**FIGURE 4:**
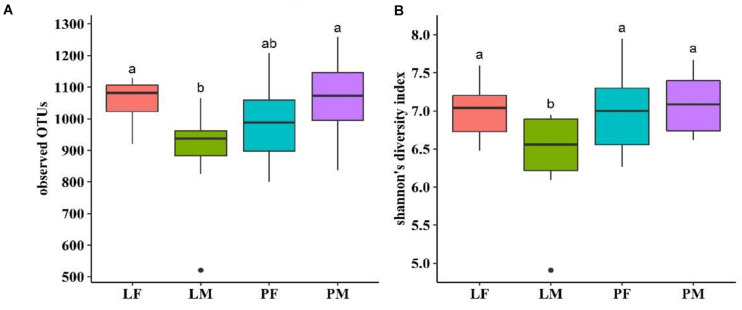
Observed OTUs **(A)** and shannon’s diversity index **(B)** of cecal microbiota in male and female ducks reared on the floor and in cages. PF, females reared on floor; PM, males reared on floor; LF, females reared in A-type cages; PF, males reared in A-type cages. Different letters indicate significant difference among groups (*p* < 0.05), same letters indicate no significant difference among groups (*p* > 0.05). *N* = 10.

Alpha diversity showed converse changes in males and females in the LY and PY groups, respectively (Figure 4). The observed OTUs and shannon indexes were significantly lower in males than females in the LY group (*p* < 0.05), while males had slightly higher values in the PY group, though the difference was not significant (*p* > 0.05).

### Beta Diversity Analysis

**FIGURE 5:**
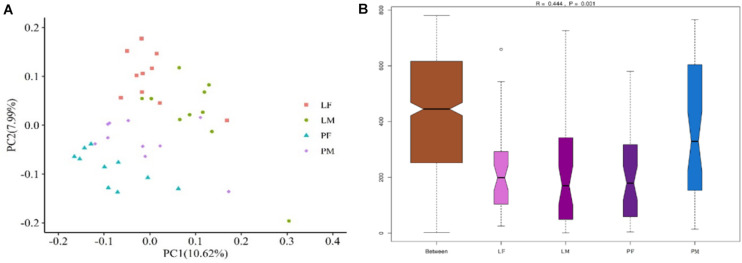
PCoA (based on unweighted UniFrac distance) **(A)** and ANOSIM analysis **(B)** of cecal microbiota in male and female ducks reared on the floor and in cages. PC1 and PC2 on x-and y-axis represent two principle discrepancy components among groups, percentage in brackets indicates contribution to the discrepancy component. Dots represent samples. Samples in same group share same color. PF, females reared on floor; PM, males reared on floor; LF, females reared in A-type cages; LM, males reared in A-type cages. *N* = 10.

PCoA was carried out using sample distance matrices, generated based on their group species phylogenic and evolutionary relationships. In unweighted UniFrac PCoA, the first principal coordinate (PC1) explained 10.62% of the variation among samples and PC2 explained 7.99% of the variation (Figure 5A). The sample dots from different rearing conditions showed distinct distances, with similar situations in males and females in the PY group, while there were no difference between males and females in the LY group. Comparisons among the groups using ANOSIM test (Figure 5B) also showed sex and feeding conditions related significant differentiation (R = 0.444, *p* = 0.001).

### Relative Abundances of Bacterial Taxa Between Different Rearing Conditions and Sexes

**FIGURE 6:**
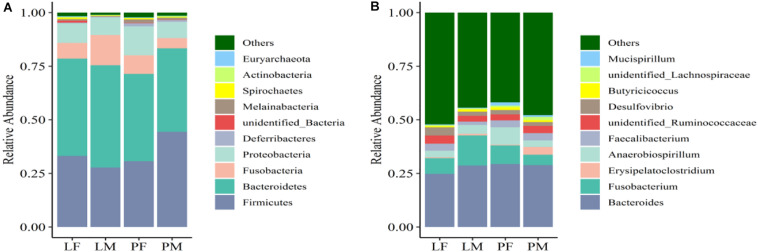
Relative abundance of cecal bacteria at the phylum **(A)** and genus **(B)** levels in male and female ducks reared on the floor and in cages. PF, females reared on the floor; PM, males reared on the floor; LF, females reared in A-type cages; LM, males reared in A-type cages.

The dominant phyla present in the cecal contents were Bacteroidetes, Firmicutes, Proteobacteria, and Fusobacteria, which represented 94.98% of OTUs (Figure 6A). The relative abundance of Bacteroidetes was significantly higher in LM compared with PM (*p* < 0.05). Firmicutes were significantly more abundant in PM and Proteobacteria in PF compared with the other groups (*p* < 0.05). The relative abundances of Firmicutes and Proteobacteria differed significantly between the sexes in the PY group (*p* < 0.05), while Fusobacteria differed between the sexes in the LY group (*p* < 0.05).

At the genus level, the relatively most abundant bacteria were *Bacteroides*, *Fusobacterium*, *Desulfovibrio*, unidentified Ruminococcaceae, *Faecalibacterium*, and *Anaerobiospirillum* (Figure 6B). The abundance of *Actinobacteria*, *Acidobacteria*, *Chloroflexi*, and *Oxyphotobacteria* differed significantly between males and females in the PY group.

### Intergroup Differences in Bacterial Species

**FIGURE 7:**
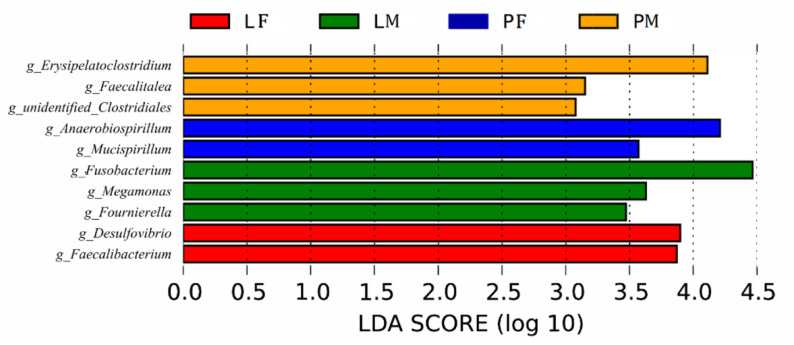
Branching diagram of the linear discriminant analysis (LDA) of cecal bacteria in male and female ducks reared on the floor and in cages. The LDA score for a discriminative feature was >3.0. PF, females reared on the floor; PM, males reared on the floor; LF, females reared in A-type cages.

LDA showed that both feeding conditions and sex affected the composition of the cecal microbiota in ducks (Figure 7): Several genera (*Desulfovibrio*, *Faecalibacterium* in group LF, *Fusobacterium*, *Megamonas*, *Fournierella* in group LM, *Anaerobiospirillum*, *Mucispirillum* in group PF, and *Erysipelatoclostridium, Faecalitalea* and *unidentified Clostridiales* in group PM) differed among the groups.

### Correlation Analysis of Differentially Detected Bacteria With Slaughter Traits and Gene Expression Levels

**FIGURE 8:**
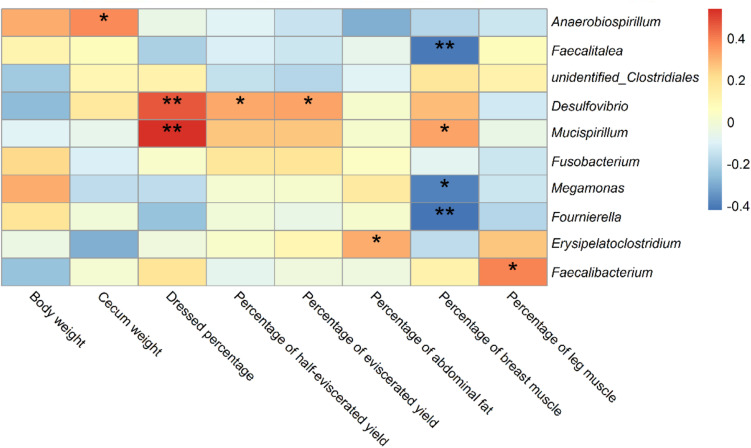
Correlations of differential bacteria genera among groups and slaughter traits in ducks. * indicates significant correlation at 0.05 level; ** indicates significant correlation at 0.01 level.

We carried out Spearman’s correlation analysis based on the relative abundance of the above bacterial genera and slaughter traits. *Anaerobiospirillum* was significantly positively correlated with cecum weight (*p* < 0.05). *Desulfovibrio* and *Mucispirillum* were significantly positively correlated with dressed percentage (*p* < 0.01). *Desulfovibrio* was also significantly positively correlated with percentage of half-eviscerated yield and percentage of eviscerated yield (*p* < 0.05). *Erysipelatoclostridium* was significantly positively correlated with percentage of abdominal fat (*p* < 0.05). *Faecalitalea*, *Megamonas* and *Fournierella* were significantly negatively correlated with percentage of breast muscle (*p* < 0.05) (Figure 8).

**FIGURE 9:**
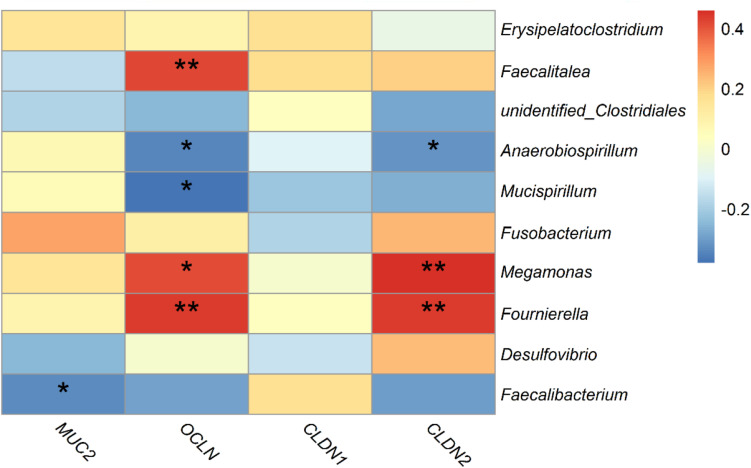
Correlations between differential bacteria genera and expression level of mucosal gene *MUC2* and intestinal structural genes *OCLN*, *CLDN1*, and *CLDN2* in duck cecum. *indicates significant correlation at 0.05 level.

Spearman’s correlation analysis was performed based on the relative abundance of the above differential bacterial genera and expression levels of intestinal mucous and structural genes. The relative abundances of *Anaerobiospirillum* was significantly negatively correlated with expression levels of the intestinal structural genes *OCLN* and *CLDN2* (*p* < 0.05). While *Megamonas* and *Fournierella* were positively correlated with expression levels of *OCLN* and *CLDN2*, respectively (*p* < 0.05). *Faecalibacterium* was negatively correlated with *MUC2* (*p* < 0.05) (Figure 9).

## Discussion

The current results revealed that rearing conditions and sex could affect the slaughter traits in ducks, although not all indicators were significantly affected in this study. As reported by Zhang et al. in Chaohu ducks, males had a significantly lower abdominal fat percentage, but there were no other significant differences between the sexes (Zhang et al., 2018). The percentage of abdominal fat was significantly higher in the LY compared with the PY group, possibly because ducks reared in cages had less exercise and were therefore more prone to abdominal fat deposition. In the current study, cecal weight was affected by the rearing conditions, with cecal weight being significantly lower in the LY compared with the PY group, while there was no significant difference between the sexes. Our findings do contradict a previous study where they found that the cecum was significantly longer in male compared with female ducks (Téguia et al., 2008). Compared with the PY groups, ducks in the LY groups were fed in A-type cages with no direct contact with feces, which may have accounted for the changes in the cecum.

In this study, we subjected ducks to different rearing conditions (rearing on the floor or on A-type cages) and kept other factors constant to examine the effects on the cecal microbiota. The results revealed that housing conditions, irrespective of nutritional formulation and other factors, affected the diversity and composition of the cecal microbiota differently between males and females. The current study revealed different numbers of bacterial OTUs among the groups. There were hundreds of unique bacterial OTUs in each group, while the numbers of unique bacterial OTUs between males and females showed opposite changes in the LY and PY groups. We speculate that the effects of rearing conditions had different effects on the cecal microbiota in male and female ducks.

Alpha diversity within the microbiota is related to health status in humans, and Le Chatelier et al. (2013) concluded that high alpha diversity was associated with good health, while low alpha diversity was related to poor health in humans. The shannon’ index was previously found to range from 4–5 among poultry (Wen et al., 2019), but varied from 7–9 in rabbits (Zeng et al., 2015), goats (Liu et al., 2014), and swine (Kim et al., 2012). The average shannon index in the current study was approximately 7, which was higher than that in chickens, and closer to that in other animals. Also in terms of the indices of observed species, the shannon index were significantly lower in males than in females in the LY group (*p* < 0.05), suggesting that rearing in cages may affect the alpha diversity within the cecal microbiota differently between sexes. The male ducks rearing on floor had slightly higher values than females. It could be because transmission between individuals have an effect on males on floor group having a smaller diversity than females. This result was consistent with the distribution of common and unique bacterial OTUs. Beta diversity analysis also illustrated differences in the microbiota composition in relation to rearing conditions and between sexes in the PY group.

The dominant bacterial phyla in the duck cecum were Bacteroidetes, Firmicutes, Proteobacteria, and Fusobacteria. In this study, the relative abundance of Fusobacteria was 4.78%–14.06% among groups, making it the fourth-dominant phylum. This represents the first report by our team of a relative abundance of Fusobacteria > 1% (Zhu et al., 2020a,b). Fusobacteria were detected in Partridge Shank chickens under free-range breeding conditions, but not in chickens fed in cages (Sun et al., 2018), while Elokil et al. reported an abundance of about 0.011%–0.060% for Fusobacteria sequenced in laying chickens housed in individual cages inside an enclosed farm (Elokil et al., 2020). These results suggest that Fusobacteria may be a normal component of the intestinal microbiota, even though some studies failed to detect its relative abundance.

The relative abundances of the most common bacteria in the duck cecum revealed that both the feeding conditions and sex affected the intestinal microbiota composition, including Bacteroidetes (LM vs PM), Firmicutes in PM, and Proteobacteria in PF. The relative abundances of the taxa also differed between the sexes, such as Firmicutes and Proteobacteria (PY group), and Fusobacteria (LY group). To the best of our knowledge, no studies to date have reported on the differences in intestinal or fecal microbiota diversity and composition between sexes in duck, although a few studies have been conducted in chicken and geese. Consistent with the results of LY groups in current study, male broiler chickens raised in wire-floored battery cages were also related to the enrichment of Bacteroides (Lee et al., 2017), while sex did not change the microbial diversity in the Greylag goose (Ricaud et al., 2019). Contrary to observations of terrestrial birds, microbiomes of Leach’s storm petrels varied mostly in relation to sex rather than environmental surroundings or social behavior (Pearce et al., 2017). Several bacterial genera were detected differentially among the groups, which could affect growth and intestinal structure in ducks. Li et al. reported that the relative abundance of *Desulfovibrio* in the cecum was effectively up-regulated to improve the growth performance and feed conversion ratio of broilers by adding probiotic *Bacillus subtilis* (Li et al., 2016). Similar results were found in weaned pigs (Xu et al., 2018). Compared with Tibetan chickens, commercial layers or Chinese domestic chickens contained more *Desulfovibrio* (Zhou et al., 2016). These reports speculated that the abundance of *Desulfovibrio* may correlate with chicken growth and performance, and that the abundance of *Desulfovibrio* could be affected by different factors, such as additives in the feed formulation, geographic position, health conditions, and genetic differences (Kováč et al., 2018). To date, there has been no similar study of the intestinal microbiota in ducks. However, the current study revealed that the relative abundance of *Desulfovibrio* was significantly different in female ducks in the LY group. Although there was no significant difference in slaughter traits between male and female LY ducks, *Desulfovibrio* was significantly correlated with the percentage of abdominal fat (R = 0.318, *p* = 0.049). Several species of *Anaerobiospirillum* were correlated with disease in other mammals and birds (Guo et al., 2018; Argüello et al., 2019; Shi et al., 2019). In this study, the feeding conditions and sex affected the abundance of *Anaerobiospirillum*, which showed a significantly higher relative abundance in female PY ducks compared with the other groups, while female PY ducks were also the lightest, and were significantly lighter than male ducks in the PY and LY groups (*p* < 0.05). Correlation analysis showed that the abundance of *Anaerobiospirillum* was significantly positively correlated with dressed percentage (R = 0.454, *p* = 0.004), percentage of half-eviscerated yield (R = 0.330, *p* = 0.040), and percentage of eviscerated yield (R = 0.324, *p* = 0.044). These results were consistent with previous reports in other mammals and poultry, suggesting that the existence and abundance of *Anaerobiospirillum* could reveal the health state of the intestine and of the host in ducks. *Erysipelatoclostridium* are opportunistic bacteria in humans. Some studies found that the relative abundance of *Erysipelatoclostridium* was significantly increased in infected animals (Wang and Wang, 2019). *Erysipelatoclostridium* was also increased in abundance in the PY group in the current study, possibly because of hygiene issues associated with the water pool. Compared with Tibetan Chicken, more *Megamonas* were contained in cecal microbiota of Lohmann layers or Chinese broiler chickens (Zhou et al., 2016). The abundance of *Megamonas* was effected by levels of feeding dietary fiber in cacal microbiota of broiler and layers (Walugembe et al., 2015). The abundance of *Megamonas, Fournierella, and Faecalitalea* were differentiated in the male groups in this study, these genera were significantly negatively correlated with percentage of breast muscle. Percentage of muscle is important economic trait of meat poultry, these genera could be candidates for regulation of the intestinal microbiota and improve productivity.

The compactness of the mucus layer is closely related to the neighboring microbiota, and microbiota structural differences within the layer were detected between germ-free and conventionally raised rats, with the colonic mucus layer appearing less compact in germ-free rats (Szentkuti et al., 1990). Bacterial overgrowth associated with bacterial translocation is linked to over-expression of *MUC2* (Vega-Magaña et al., 2018). In the present study, *MUC2* expression levels were slightly higher in the PY group and significantly higher in the PM compared with LF group, there was a negative correlation between *Faecalibacterium* and *MUC2* gene expression level. Tight junctions are the most important structural component of a constitutive epithelial cells barrier, by regulating barrier permeability via tight sealing of cell-cell junctions. Tight junction proteins are represented by claudins, occludin, junctional adhesion molecules, and zonula occludens scaffold protein (Oshima and Miwa, 2016). Disruption of the intestinal tight junction barrier induces perturbation of the mucosal immune system and inflammation, and can act as a trigger for the development of intestinal and systemic diseases (Suzuki, 2013). The tight junctions in the intestine could be affected by the neighboring cecal microbiota. Several bacterial genera were significantly positively related to the expression levels of tight junction barrier genes (*OCLN*, *CLDN2*), including *Faecalitalea, Megamonas* and *Fournierella.*

In conclusion, the dominant bacterial phyla in the duck cecum were Bacteroidetes, Firmicutes, Proteobacteria, and Fusobacteria, with Fusobacteria being the fourth-dominant phylum. We demonstrated that both rearing conditions and sex could affect the cecal microbiota diversity and composition. Several bacteria genera showed differential abundances among the groups, the correlation analysis suggested that these bacterial taxa could affect the growth and intestinal structure in ducks. Battery rearing technology has been developed for ducks to improve animal health and environmental protection. Understanding the differences in intestinal microbiota among different rearing patterns and sexes will facilitate targeted monitoring and regulation of the intestinal microbiota to further ensure the health and productivity of the domestic duck population.

## Data Availability Statement

The original contributions presented in the study are publicly available. This data can be found in NCBI, under accession number PRJNA655698.

## Ethics Statement

The animal study was reviewed and approved by the Committee of Animal Care at Jiangsu Institute of Poultry Science (CAC-JIPS01453, Yangzhou, China).

## Author Contributions

CZ, WX, and HuL conceived, designed the study, and wrote the manuscript. WX and ZT performed the experiments. WS and HoL analyzed sequencing data and experimental results. HoL also assisted with amplicon sequencing. HuL obtained project funding and wrote the manuscript. SZ, CZ, and WX reviewed and edited the manuscript. All authors read and approved the manuscript.

## Conflict of Interest

The authors declare that the research was conducted in the absence of any commercial or financial relationships that could be construed as a potential conflict of interest.

## Footnotes

1 http://www.drive5.com/usearch/manual/uchime_algo.html
